# Identification of Temperature-Induced Deformation for HSR Slab Track Using Track Geometry Measurement Data

**DOI:** 10.3390/s19245446

**Published:** 2019-12-10

**Authors:** Zai-Wei Li, Xiao-Zhou Liu, Yue-Lei He

**Affiliations:** 1 School of Urban Rail Transportation, Shanghai University of Engineering Science, Shanghai 201620, China; zaiweili@sues.edu.cn (Z.-W.L.); hyldoc@163.com (Y.-L.H.); 2 Institute of Urban Smart Transportation & Safety Maintenance, Shenzhen University, Shenzhen 518060, China

**Keywords:** high-speed rail (HSR) slab track, slab-warping deformation (SWD), track geometry data, wavelet-based feature extraction

## Abstract

Slab track is widely used in many newly built high-speed rail (HSR) lines as it offers many advantages over ballasted tracks. However, in actual operation, slab tracks are subjected to operational and environmental factors, and structural damages are frequently reported. One of the most critical problems is temperature-induced slab-warping deformation (SWD) which can jeopardize the safety of train operation. This paper proposes an automatic slab deformation detection method in light of the track geometry measurement data, which are collected by high-speed track geometry car (HSTGC). The characteristic of track vertical irregularity is first analyzed in both time and frequency domain, and the feature of slab-warping phenomenon is observed. To quantify the severity of SWD, a slab-warping index (SWI) is established based on warping-sensitive feature extraction using discrete wavelet transform (DWT). The performance of the proposed algorithm is verified against visual inspection recorded on four sections of China HSR line, which are constructed with the China Railway Track System II (CRTSII) slab track. The results show that among the 24,806 slabs being assessed, over 94% of the slabs with warping deformation can be successfully identified by the proposed detection method. This study is expected to provide guidance for efficiently detecting and locating slab track defects, taking advantage of the massive track inspection data.

## 1. Introduction

In past few decades, the development of high-speed rail (HSR) has been astonishing in many countries. In China, for instance, the operating mileage of high-speed railway networks will reach 31,000 km by the end of 2019, with around 4000 in-service high-speed electric multiple units (EMUs) running on the world’s longest HSR network, carrying over 6 million passengers every day. To ensure high quality, reliability, and efficiency in HSR operation, slab track, characterized with good track alignment and low maintenance requirement, has found extensive application.

The design of HSR slab track has been improved a lot since 1970s (earlier slab track systems constructed in Germany and Japan [1,2]), which made it successful in HSR applications, especially in Japan and China where the newly built lines include a high percentage (up to 96 %) of slab track [3]. Nevertheless, some problems inevitably occur in track substructures due to the coupling effect of the trainload and environmental actions [4,5,6,7]. In particular, the HSR network covers a vast territory, and slab track is constructed all over the world at places with different weather conditions, e.g., great annual temperature difference, great daily temperature difference, and continuous high temperature [8,9]. On-site observations of in-service HSR lines show that deterioration in slab tracks and the structural damages are frequently reported on the substructures of slab track during the high-temperature period [8]. Such damages may reduce the constraint between different layers of slab tracks, increase track irregularity level, and induce abnormal vibration of the vehicle–track system [9]. If structure damages remain undetected and unrepaired, they may eventually cause complete fracture of the track system and jeopardize operational safety. Thus, it is highly desirable to understand the mechanism and effect of slab defects and to keep substructures of the track in satisfactory in-service states and resistant to unplanned failure.

To investigate how track slab damages initiate and how they affect vehicle–track system dynamics and track irregularities, previous studies carried out numerical simulations [3,4,5,6,7,8,10,11,12,13,14,15,16], full-scale tests [9,17,18,19], and field tests [15,20,21]. These studies shed some light on repair works for defects, slab maintenance, and life-cycle management of slab track. However, the performance of slab track is spatially and temporally variant and is affected by numerous factors so that the damages can occur unexpectedly. In fact, tremendous efforts have been spent on daily inspection and maintenance of slab track to ensure safe and reliable operation. Due to the lack of information about the state of the slabs during their operation, the inspections are scheduled periodically on time or kilometer basis, which incurs high inspection and maintenance costs. Therefore, there is a clear need and a large economic incentive for developing advanced detection methods that can effectively locate defective slabs, identify the structural damages, and determine maintenance and repair needs.

A straightforward method of damage detection for slab track is regular visual inspection or non-destructive testing (NDT) methods [20,21,22,23,24,25,26,27,28]. The NDT techniques are powerful in damage detection for concrete structures, especially in structural health monitoring of bridges [29,30,31]. The NDT techniques adopted in ballastless railway detection mainly includes impact-echo method [21,23,24], ground-penetrating radar (GPR) [21], digital image correlation (DIC) [25,26] and synthetic aperture focusing technique (SAFT) ultrasound imaging method [27,28]. They can detect specific slab defects and report the size and even geometry of defects through customized devices. However, the HSR network is of very large scale and has the strict requirement of track alignment, so the conventional inspection methods are no longer feasible because they are time-consuming and claim long possession of window time. The narrow time windows for track maintenance in HSR calls for an efficient method for massive slab inspection and detection of potential defective slabs that need to receive maintenance or repair. On the other hand, the maintenance-of-way department has a large quantity of track geometry measurement data, but little has been done with the data other than generating reports of track geometry condition. Many existing studies concentrated on analysis of track geometry data to access the track quality at different operation sections. As regards the problem of extracting and locating track slab defects, however, the studies are rather scant.

This paper proposes a novel detection method for slab-warping deformation (SWD) taking advantage of track geometry data. The main challenge is how to extract the SWD-sensitive feature. In this paper, the feature of slab deformation on track geometry data is first observed in both time and frequency domain and by time–frequency analysis. On this basis, wavelet decomposition is employed for extracting signatures or features out of the track irregularity signals. An index for slab condition assessment, named slab-warping index (SWI), is then defined. The algorithms in the detection procedure are coded in MATLAB environment so that the defective slab can be located automatically. The aim of this paper is to enable decision-making on the maintenance for slab. It is worth mentioning that the raw track geometry data contain irrelevant information, noise, and position errors, so the proposed method includes a positional synchronization method in data preprocessing and a discrete wavelet transform (DWT)-based feature extraction method, both of which can enhance the accuracy of locating potential SWDs and assessing their severity. This work is among the efforts toward solving a scientific problem: How to identify a defect with both high efficiency and accuracy for large-scale structures like railways.

The rest of this paper is outlined as follows. The warping deformation of HSR slab is introduced in Section 2. Section 3 presents track geometry measurement with high-speed track geometry car (HSTGC) and the proposed track slab-warping deformation identification procedure. The in situ verification of the procedure for HSR slab track is presented in Section 4. Finally, some conclusions are given in Section 5.

## 2. Temperature-Induced Slab Defects and Warping Deformation

Slab track is constructed all over the world in places with different weather conditions and temperature and is a critical factor that can influence the behavior of slab track systems, especially for track slab [8,18,32] and cement-emulsified asphalt (CA) mortar [33,34,35,36,37]. Due to the effect of solar radiation in daytime, the temperature at the top of the track slab is higher than that at the bottom, thereby leading to temperature gradient load [13,38]. It was found by Zhu and Cai [6] that the temperature difference between the top and bottom surfaces of track slab can reach over 16 °C. The temperature gradient load induced by the great temperature differential may result in the generation of pulling and curling stresses in the prefabricated concrete slabs [32] and structural defects. Typical temperature-induced slab track defects include interface debonding/delamination between CA mortar and track slab [4,7,12,18,25,26,32,39], decreased compressive strength, cracking and damage of CA mortar [9,15,16,33,35,40,41,42], wide and narrow juncture defects [8], warping deformation of track slab [13,16,26,38], etc.

**Figure 1:**
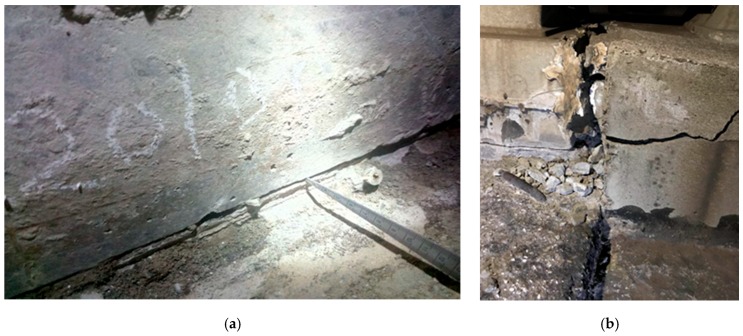
Temperature-induced slab-warping deformation (SWD): (**a**) interface debonding of concrete slab due to SWD; (**b**) damage of joint concrete due to SWD.

Among these track defects, warping deformation of slabs (Figure 1) attracts much attention from HSR operators as it can not only deteriorate the mechanical properties of slab track but also influence the geometric condition of slab track [16]. Warping deformation starts with initial misalignment/upward curl during the construction phase and usually occurs during high-temperature seasons [16]. Under the aforementioned temperature effect, the top surface of slabs will elongate, while the bottom will compress [16,20,26,39]. As a result, slab warps upward due to eccentric compression. Field surveys of SWD at some HSR lines showed that warping of slab occurred preferably around broad joint, and warping deformation of the coupled slab at bridge is measured to be 5 mm from its initial configuration on average, up to 20 mm in extreme cases [16]. It is thus clear that this is an urgent problem to be solved from the viewpoint of the safety operation of HSR.

## 3. SWD Detection Based on Track Geometry Data

Compared with the conventional inspection methods for HSR slab tracks, in which it is impossible to inspect/monitor infrastructure conditions at every track segment, detection methods based on track geometry data provide opportunities to investigate infrastructural conditions for the whole line or even the whole HSR network. Through track geometry data, the state of rail infrastructure can be observed at inspection times in an exact and efficient way. By analyzing the geometry condition of the same track segment over and over again in different inspection runs, one can observe track deterioration including warping deformation through track geometry changes [43]. Therefore, it is reasonable to mine the track geometry data and extract the deterioration-sensitive features for the identification of defective slabs, especially for warping deformation detection.

### 3.1. Track Geometry Data Collected by High-Speed Track Geometry Car (HSTGC)

**Figure 2:**
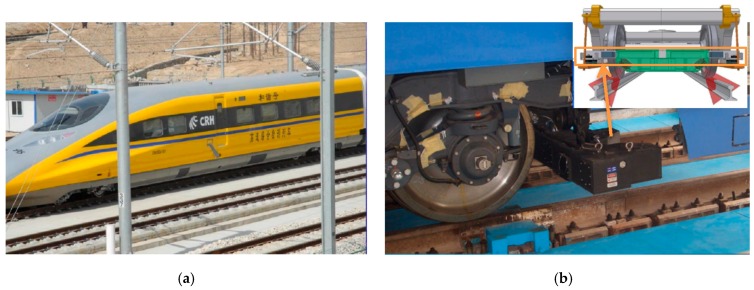
(**a**) Chinese Comprehensive Inspection Train (CIT) for high-speed rail (HSR) infrastructure inspection; (**b**) track geometry measurement system.

Track geometry condition is periodically measured with track geometry car (TGC), track geometry trolley, and some other manual tools [44]. Among these tools, TGC, which conducts measurement under wheel loading conditions, offers high efficiency in reporting track irregularities. Nowadays, there are many types of high-speed track geometry cars (HSTGCs) running on the HSR network in different countries. They can conduct infrastructure inspection at high speed (>200 km/h), and the track geometry information of HSR network can be grasped by a few HSTGCs. Figure 2 shows an example of a HSTGC named No. 0 Comprehensive Inspection Train (CIT) developed by China Academy of Railway Sciences. Its inspection covers all HSR lines (>30,000 km) in the Chinese national HSR network. At each inspection run, the HSTGC measures track dynamic irregularities and ride comfort parameters (vehicle vibration) at each sampling point along the rail line [43]. The dynamic irregularities include: Gauge, left/right alignment, left/right surface, cross-level, and twist [22,44,45,46]. The measurement system consists of different measuring units, which measure different irregularities based on specific sensing techniques: Gauge is measured based on laser photography, alignment and surface are measured with inertial reference unit, cross-level (cant) is measured by gyro platform and displacement sensors. Track geometry can therefore be described in terms of track curvature, alignment, elevation (cant), and gauge as functions of distance along the track [47].

With the measurement data of track irregularities, track condition can be assessed. To quantify the track condition, there are indexes defined based on track geometry data by different countries, including SNCF’s Mean Deviation Indices (France), Chinese Track Quality Index (TQI), SD index (UK, Australia, etc.), Q Index (The Netherlands), P Index (Japan), Track Roughness Index (TRI, America Amtrak), Track Geometry Index (TGI), etc. Most of these indexes are obtained by calculating the standard deviations of geometry data [22] and mainly focus on track quality assessment for a long section (e.g., 200 m), while they are not sensitive to local defects such as SWD, the sensitive wavelength of which is only a few meters (the slab length).

In fact, besides being used to assess infrastructure condition, track geometry data contain highly valuable information, which can reflect potential local defects of track substructures, such as rails, fasteners, slabs, concrete bases, and even subgrade/bridges/tunnels. With recognition of this, we propose a data-driven slab deformation detection method in light of track geometry data. In the following sections, a signal preprocessing technique is used to eliminate milepost error and outliers of the raw track geometry data. The feature of SWD is then analyzed in both time and frequency domain, followed by the establishment of slab-warping index (SWI) for warping deformation assessment using discrete wavelet transform (DWT)-based feature extraction technique. Based on the fact that SWD mainly influences the track geometry in the vertical direction, this study mainly focuses on the analysis of track vertical irregularity data.

### 3.2. Data Pre-Processing: Eliminating Milepost Error

Despite HSTGC representing the cutting-edge technology in rail infrastructural condition inspection, a chance of measurement error due to various factors cannot be completely eliminated. Among these errors, milepost positional error [43,48] is a critical one, especially when there is a need to locate the track segment corresponding to anomalies on the waveform of track geometry data. In practice, the mileage information is obtained from the rotation angles and the wheel radius. However, positional errors can inevitably occur and accumulate due to radial errors of the wheels, faulty encoder output, degraded adhesive conditions, and track geometry irregularities [48]. Field investigations have found that the milepost position could be off up to 200 m [43], which is much longer than the length of track slab. With uncorrected milepost error, the defective slabs are difficult to locate. Therefore, eliminating milepost error before further analysis is a critical task in slab deformation detection based on track geometry data. This paper employs grey incidence analysis (GIA), also called grey relational analysis (GRA) [49] to correct milepost errors of track geometry data. The basic idea of GIA is to quantify the degree of similarity of the geometric curves of different data sequences [49].

To perform milepost error correction, we first correct absolute position errors with field milepost information and obtain a standard geometry dataset from a set of inspection data, denoting as a reference vector in GRA: **X_0_** = {*x*_0_ (*j*)}, (*j* = 1, 2,... , *n*). With the standard geometry data, the data collected by other inspection runs will be subjected to GRA-based position synchronization. Because the correction process is to translate the waveform of geometry data to match the standard data, the length (denoted as *N*) of geometry data sequences subjected to synchronization should be larger than *n*, and the number *m* = (*N* − *n*)/2 is the maximum one-side translation quantity in the correction process. As such, we can define the geometry data sequence to be corrected **Y** = {*y*(*k*)}, (*k* = 1, 2,..., *N*, *N* > *n*) and the subsequences of **Y** in GRA process **Y*_i_*** = {*y_i_*(*j*)}, (*i* = −*m*, ..., 0, ..., *m*, *j* = 1, 2,..., *n*), where

(1) $$\begin{array}{c} Y_{-m}=\left\{y\left(1-m+\frac{N-n}{2}\right),y\left(2-m+\frac{N-n}{2}\right),\cdots ,y\left(n-m+\frac{N-n}{2}\right)\right\}\\ \cdots \cdots \\ Y_{i}=\left\{y\left(1+i+\frac{N-n}{2}\right),y\left(2+i+\frac{N-n}{2}\right),\cdots ,y\left(n+i+\frac{N-n}{2}\right)\right\}\\ \cdots \cdots \\ Y_{m}=\left\{y\left(1+m+\frac{N-n}{2}\right),y\left(2+m+\frac{N-n}{2}\right),\cdots ,y\left(n+m+\frac{N-n}{2}\right)\right\} \end{array}$$

According to GRA, the degree of grey incidence between **X_0_** and **Y*_i_*** is

(2) $$\gamma_{i}=\frac{1}{n}\overset{n}{\underset{j=1}{\sum }}\gamma \left(x_{0}\left(j\right),y_{i}\left(j\right)\right)$$

where *γ*(*x*_0_(*j*), *y_i_*(*j*)) is the incidence coefficient which is defined as

(3) $$\gamma \left(x_{0}\left(j\right),y_{i}\left(j\right)\right)=\frac{\underset{i}{min}\underset{j}{min}\left|x_{0}\left(j\right)-y_{i}\left(j\right)\right|+\rho \underset{i}{max}\underset{j}{max}\left|x_{0}\left(j\right)-y_{i}\left(j\right)\right|}{\left|x_{0}\left(j\right)-y_{i}\left(j\right)\right|+\rho \underset{i}{max}\underset{j}{max}\left|x_{0}\left(j\right)-y_{i}\left(j\right)\right|}$$

where *ρ* is known as the distinguishing coefficient and *ρ* ∈ (0,1).

**Figure 3:**
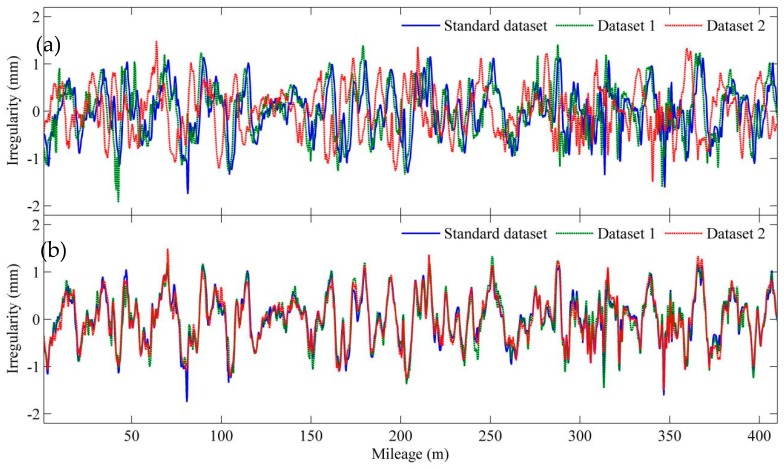
Milepost error correction: (**a**) waveform of standard geometry and track geometry datasets before position synchronization; (**b**) waveform of geometry and track geometry datasets after synchronization.

With Equation (2), we can obtain 2*m* + 1 degrees of grey incidence between **X_0_** and different subsequences of **Y**, denoted as **Γ** = {*γ_i_*} (*i* = −*m*, ..., 0, ..., *m*). Then the translation of geometry dataset **Y** in position synchronization can be determined by taking the maximum element of the sequence **Γ**:

(4) $$T\left(X_{0},Y\right)=argmax_{i\in \left[-m,m\right]}\gamma_{i}$$

where *T*(**X_0_**,**Y**) is the translation distance of geometry dataset **Y** to match standard dataset **X_0_** in position synchronization. Figure 3 shows the result of position synchronization for two sets of track vertical irregularity data (where *ρ* = 0.1). It is seen that the milepost errors, which are defined as the differentials between the two datasets to be synchronized and the standard irregularity data, are 4 m and 10 m, respectively. After position synchronization, the milepost errors of both datasets are successfully eliminated and the corrected irregularity datasets can be used for slab deformation detection, as shown in Figure 3b.

### 3.3. Analysis of Slab-Warping Feature on Track Geometry Data

#### 3.3.1. Time-Domain Analysis

**Figure 4:**
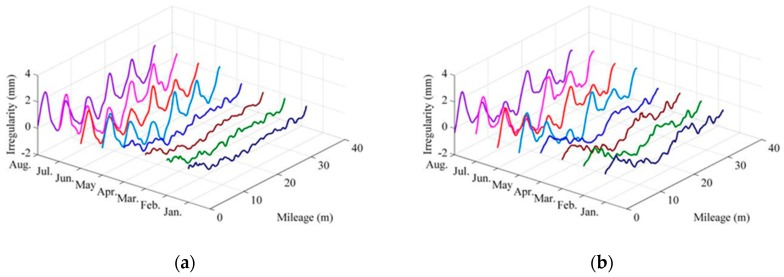
Track vertical irregularities of an HSR line with temperature-induced SWD: (**a**) left rail; (**b**) right rail (Note: The atmospheric temperatures at the date of inspection from January to August are −1, 6, 10, 18, 27, 28, 36, 32 °C, respectively).

As aforementioned, the track geometry measurement is usually carried out every month by HSTGC, so that the change of track alignment condition can be observed. More importantly, the deterioration trend and performance of maintenance work (if any) can be investigated. Figure 4 shows track vertical irregularity measurement results from January to August of a year for a section of rail line that suffers from temperature-induced SWD. It is seen that: (1) the left and right vertical irregularity generally present similar variation trends with mileage; (2) the instability of vertical irregularity starts from May and achieves maximum peak–peak value in July and August; (3) the vertical irregularity has a strong correlation with atmospheric temperatures (i.e., the effect of high temperature is obvious).

Through time-domain analysis, the feature of temperature-induced SWD can be found on the waveform of track vertical irregularity data. However, sometimes the amplitude of track vertical irregularity at the slabs with SWD is still below the manufacturing/maintenance tolerance limit, which means it is difficult to detect SWD from massive track geometry data with time-domain analysis only.

#### 3.3.2. Frequency-Domain Analysis

**Figure 5:**
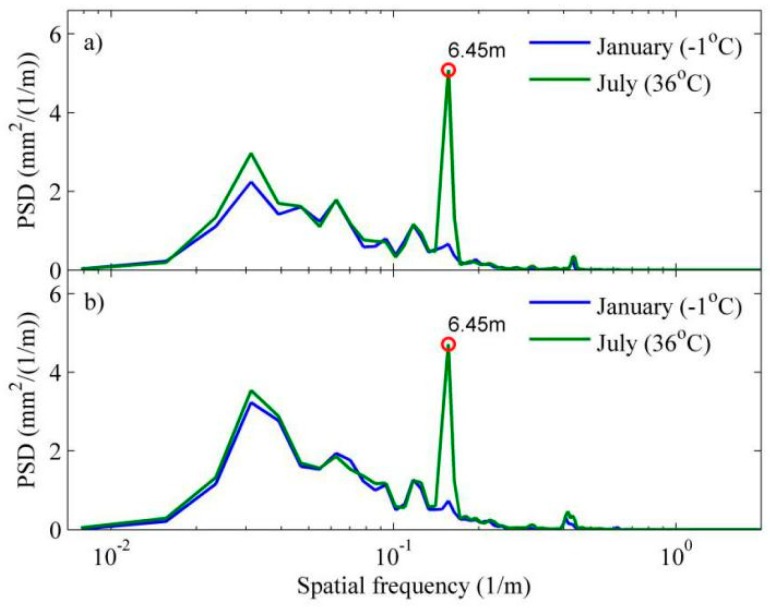
Power spectrum densities (PSDs) of track vertical irregularity data samples: (**a**) left rail; (**b**) right rail.

Since temperature-induced SWD generally occurs on the longitudinal direction, and the length of track slab is constant, the feature wavelength of SWD signature on track geometry data is expected to be equal to the length of track slab. As such, the SWD-sensitive feature may be captured through frequency-domain analysis for track geometry data. In this paper, power spectrum densities (PSDs) of vertical irregularity data are calculated based on Welch’s method, in which the Hann window is employed. Figure 5 shows the PSDs of left and right vertical irregularities. The two samples of track geometry data were collected by two inspection runs when the atmospheric temperatures were −1 °C (in January) and 36 °C (in July). The track slab of the section being inspected is longitudinally coupled slab, the unit length of which is 6.45 m.

It is seen that: (1) the distributions of PSDs of both left and right irregularities are roughly the same—multiple peaks are observed with only a slight difference in amplitudes, which indicates there are periodic irregularities of track structure; (2) comparing two geometry data samples, a big difference in PSD amplitude at 6.45 m wavelength is found, which indicates that irregularity at the wavelength equal to slab length increases rapidly as temperature rises. Through frequency-domain analysis, the feature of potential SWD can be revealed. However, PSD of track irregularity is calculated for a section of track (e.g., 1024 m), and the length is usually much longer than slab length. Therefore, with PSD, we can only recognize some track sections that may have warped slabs but cannot locate these slabs and quantify the severity of SWD.

#### 3.3.3. Time–Frequency Analysis by Discrete Wavelet Transform (DWT)

Based on time and frequency-domain analysis in Section 3.3.1 and Section 3.3.2, the signature of SWD on vertical irregularity measurement data can be characterized. However, as aforementioned, both time-domain analysis and frequency-domain analysis are incapable of locating warped slabs and quantifying the severity of SWD. Track irregularity data can be transient/non-stationary, especially in cases where the inspection section covers anomalies (e.g., potential SWD or other deterioration of track infrastructure). In this regard, the time–frequency analysis technique should be adopted. DWT, as a common time–frequency analysis technique, is developed to decompose a signal into a set of orthonormal bases that correspond to different time and frequency scales or resolutions with varying frequency bandwidths [50,51]. At each level of decomposition, approximation and detail coefficients can be obtained from the approximation coefficients of previous decomposition, or original signal if it is first-level decomposition.

**Figure 6:**
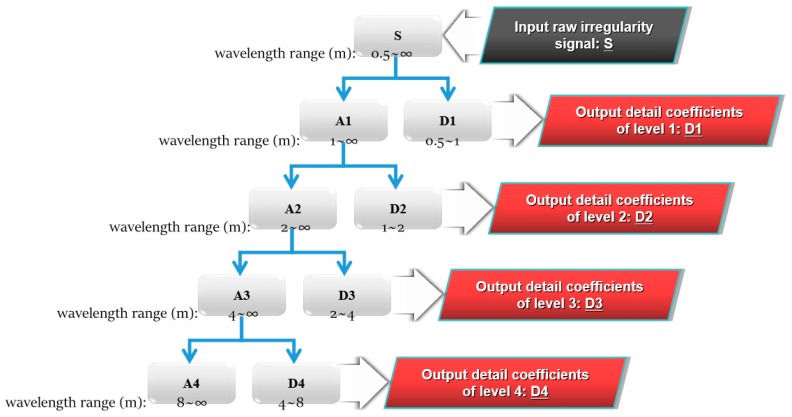
A typical four-level discrete wavelet transform (DWT) for track geometry data (sampling interval = 0.25 m).

In this study, we adopt multi-level wavelet decomposition based on Mallat’s algorithm for extracting signatures or features out of the track vertical irregularity signals. Based on the fact that Daubechies wavelets provide both compact-support and regularity, the DB4 wavelet is selected in DWT. Then we can obtain the detail coefficients at all levels, denoted as signals D*j* in the order of increasing spatial frequency (descending wavelength). The feature of SWD can be found in detail coefficients of a certain level that correspond to a wavelength range covering the length of slab being detected. The cut-off wavelengths of signal D*j* can be estimated as

(5) $$\begin{array}{l} Lu_{j}=\frac{2^{j+1}}{fs}\\ Ll_{j}=\frac{2^{j}}{fs} \end{array}$$

where *Lu_j_* and *Ll_j_* are upper and lower limits of *j*th wavelength range; *fs* is the spatial sampling frequency. Figure 6 shows an example of a four-level DWT process and the wavelength ranges of detail coefficients of all the levels, provided that the sampling interval of HSTGC is 0.25 m (*fs* = 4).

**Figure 7:**
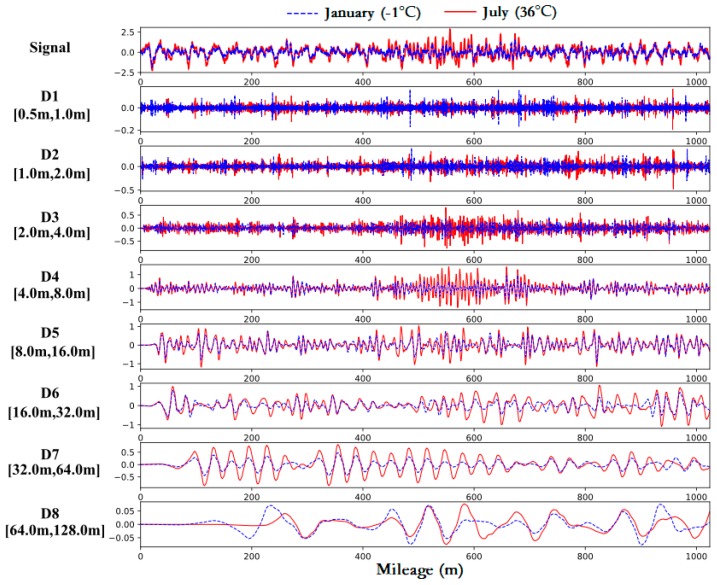
The original signal of vertical irregularity and its detail coefficients at all levels after DWT.

Figure 7 shows the result of eight-level DWT using Mallat’s algorithm. The samples used in the analysis are the same as those used in frequency-domain analysis. As can be seen from Figure 7, the anomalies can be found in section 500–600 m on the raw signal collected in summer. After DWT, the anomalies are mainly present in D4 corresponding to wavelength range of 4–8 m. The length of slabs being detected is 6.45 m, which is within this range. Therefore, the feasibility of extracting slab-warping features and locating the warped slabs by DWT is corroborated.

### 3.4. Slab-Warping Deformation Assessment

A primary goal of this paper is to make a rational decision about whether a track slab should be repaired (i.e., to establish a condition-based maintenance (CBM) scheme for track slab). Similar with CBM for vehicle components using data collected by track-based detectors [52,53], the requirement of CBM for track infrastructure in light of vehicle-based inspection also means a need for a signal processing algorithm that can automatically extract the feature of defects (the defect discussed in this paper is SWD). Based on the analysis of slab-warping feature in Section 3.3, this section employs DWT to detect and locate the slabs with SWD. On this basis, a slab-warping index (SWI) is established for quantifying the severity of warping deformation of track slab.

#### 3.4.1. Feature Extraction and Establishment of SWI

As discussed in Section 3.3, the DWT has shown its potential in SWD detection. Therefore, to quantify the severity of SWD, we adopt the DWT-based feature extraction method for extracting salient features of warped slabs out of the track irregularity signals. Based on the analysis results in Section 3.3.3, we use right-level wavelet decomposition and select the detail coefficients of level *j* in DWT D*j* as the bases for performing feature extraction. Note that *j* is determined by the slab length, i.e., to search the reconstructed signal (from D1 to D8), which the corresponding wavelength can cover, the slab length and the wavelength range of each level is obtained by Equation (5). Then, to quantify the severity of SWD, SWI is established, which is defined as the root mean square (RMS) of reconstructed signal D*j*:

(6) $$SWI=\sqrt{\frac{\sum_{i=1}^{n}s_{\left(j\right)i}{}^{2}}{n}}$$

where *s*_(*j*)*i*_ is the *i*th data point in the reconstructed signal (sequences), which is obtained by DWT presented in previous Section, and *n* is the sample size in SWI calculation, and

(7) n=fs⋅L

where *fs* is the spatial sampling rate of TGC in data collection and *L* is the length of track section subject to SWI calculation. It is suggested that *L* should be larger than slab length *l* to ensure the deformation signature can be fully captured, while smaller than 2*l* so that the condition of each slab can be examined individually.

**Figure 8:**
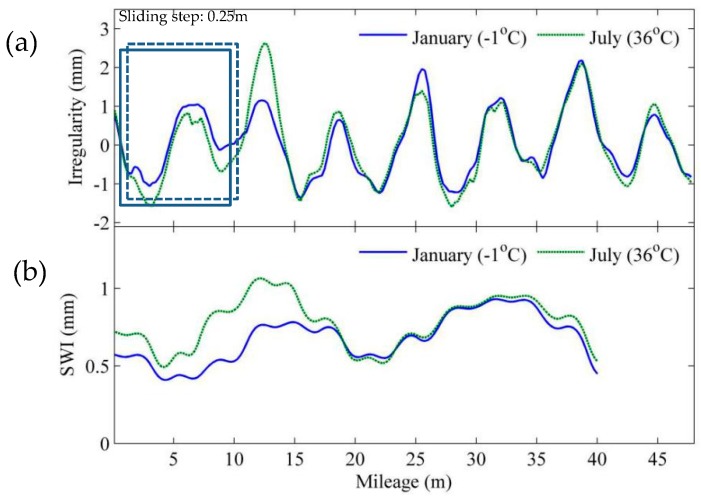
Slab-warping index (SWI) calculation: (**a**) moving window on geometry data; (**b**) calculation results.

However, it is still difficult to locate a single track slab on the waveform of geometry data, though position synchronization is employed in data prepossessing. In this regard, we adopt the moving window approach in SWI calculation, as illustrated in Figure 8. The window starts from the first element and keeps shifting right by one element each time (sliding step is equal to the sampling interval), and the SWI is calculated every step. With the moving window, an SWI sequence can be obtained, the length of which is *N* − *n* +1, where *N* is the sample size of input geometry data, and *n* is the size of the window, which is the same as the *n* in Equation (7) (sample size of data subjected to every step of SWI calculation).

#### 3.4.2. Slab-Warping Deformation Identification

**Figure 9:**
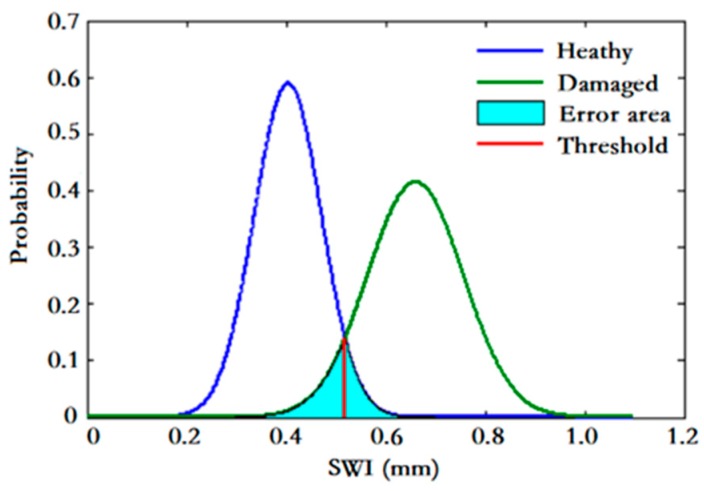
Pattern classification.

**Figure 10:**
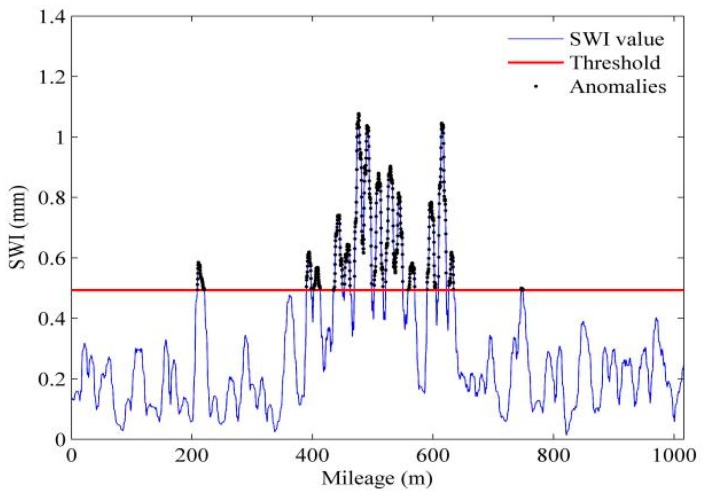
Identification of slabs with warping deformation by SWIs.

With the SWI, the slab condition in terms of warping deformation can be assessed. However, the question remains about how to determine whether a slab has SWD based on the values of SWIs. To provide an accurate classification, a threshold should be defined for discrimination between healthy and warped states of slab. Normally, it is determined by a pattern classifier, which is formulated in terms of minimum error rate classification. The threshold is set at the intersection of the two probability density functions (PDFs) of SWIs from healthy and warped respectively, as illustrated in Figure 9. The overlap area shown in the figure indicates the error rate in decision making. Once the threshold is set, the slabs with SWD can be identified by checking whether the SWIs exceed the threshold, as shown in Figure 10.

**Figure 11:**
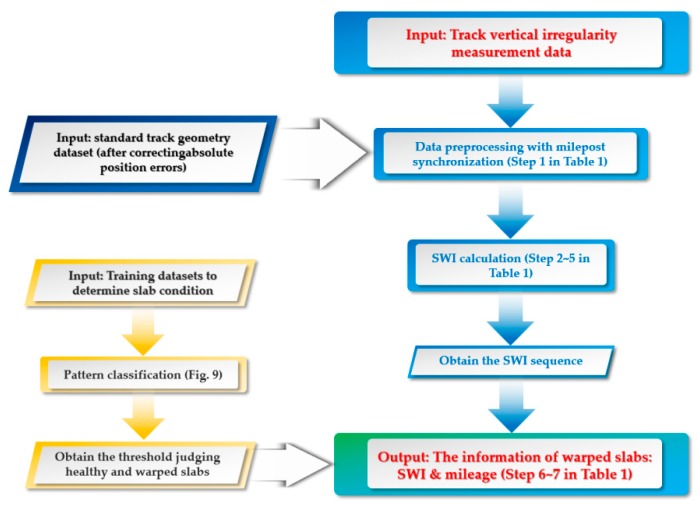
The procedure of SWD identification.

Summarizing, the SWD calculation is a four-step process, as shown in Table 1. Figure 11 shows the whole procedure of SWD identification. With this procedure, the segments with warped slabs can be detected along with the severity of SWD automatically, thereby the slab maintenance can be carried out accordingly.

## 4. Case Study: Warping Deformation Detection for CRTS II Slab

In this section, the capability of detecting SWD of the proposed method is verified through a blind test. In this test, two double-tracked HSR sections constructed with China Railway Track System (CRTS) II slab track are selected as the testbed. This type of slab track system is briefly introduced in Section 4.1. These rail sections are located at places where the maximum air temperatures can rise up to 40 °C in summer.

Before the test, the threshold to judge healthy and warped slabs is trained with two sets of data containing both track geometry data and track defect database, as detailed in Section 4.2. It should be noted that the defect database is recorded by the maintenance-of-way department, which is carried out by periodic visual inspection because the slab track deteriorates through their lifetime, and temperature-induced defects occur frequently. By comparing the detection results with the regular visual inspection results, the performance of the proposed method is evaluated. The detection results and performance analysis of the proposed slab-warping detection method are detailed in Section 4.3.

### 4.1. CRTSII Slab Track

**Figure 12:**
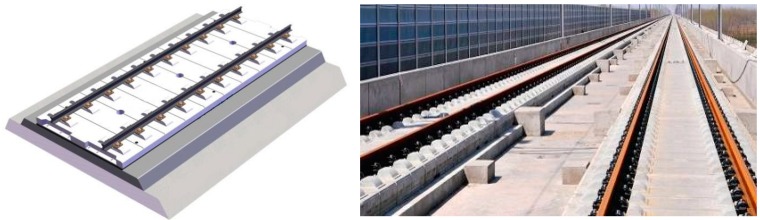
China Railway Track System II (CRTSII) slab track.

CRTS II slab track is one of the most widely used ballastless tracks in China HSR. It is applied in more than 10 HSR lines, including Beijing–Shanghai HSR, Nanjing–Hangzhou HSR, Shanghai–Kunming HSR, etc., [18]. As a prefabricated slab track system (prefabricated in factory), it is technically improved from the Bögl slab track [12]. The track components from top to bottom are CN60 rail, fastener, track slab, CA mortar layer, and concrete base, as shown in Figure 12.

The prefabricated slab is 20 cm thick, 6.45 m long, and 2.55 m wide, and concrete strength grade is C55. As a longitudinal continuous structure, the slabs are connected through the joint between slabs [8] and are bonded to concrete base (C15 concrete cast in place, 30 cm thick, 6.80 m long, and 3.30 m wide) firmly by CA mortar layer (30 mm thick) [18], which is injected afterwards to serve as a cushion layer and shock-absorber [39,41,42].

Several on-site observations show that in the high-temperature season, the prefabricated slabs are prone to warping deformation, which can further cause damages of joint concrete, debonding of the CA mortar layer, etc.

### 4.2. Test Condition

In this study, track geometry is measured by the Chinese new generation of Comprehensive Inspection Train (CIT), the type of which is CRH380AJ, a re-vamped commercial trains specifically designed for HSR inspection. The HSR line, constructed with CRTSII, is located in eastern China, a region with high-temperature record (>35 °C) every year. The No. 0 CIT conducted routine inspection for the rail track once a month. Among the inspection data, the vertical irregularity data, collected at a sampling interval of 0.25 m, are used for SWD identification.

As aforementioned, before identifying the SWD, the threshold needs to be set. The determination of the threshold is presented in Section 3.4.2. The training data used in this paper are collected from both healthy slabs and warped slabs. As shown in Table 2, there are 120,110 and 625 track geometry data samples corresponding to healthy slabs and slabs with SWD, respectively. With the proposed threshold determination method, the limit of SWI for judging whether a slab is healthy or warped is set as 0.4932 (unit: mm), with an error rate of 2.3% (type I error rate: 1.09%, type II error rate: 1.21%).

### 4.3. Test Results and Validation

**Figure 13:**
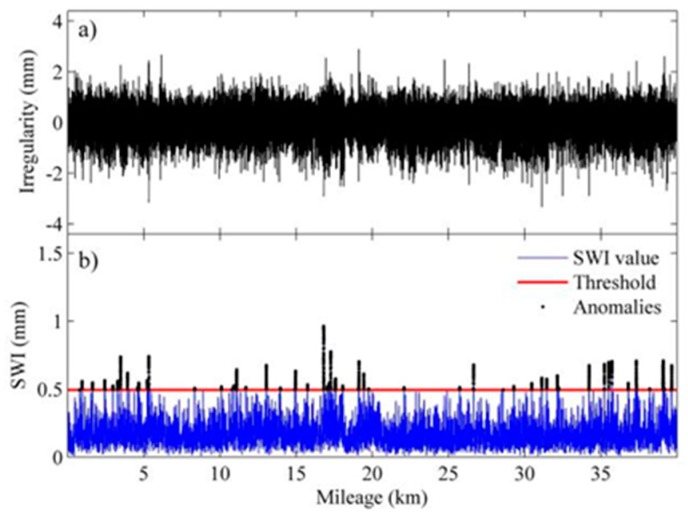
Track geometry and SWI of rail Section I: (**a**) waveform of track vertical irregularity; (**b**) SWI sequence and anomalies.

**Figure 14:**
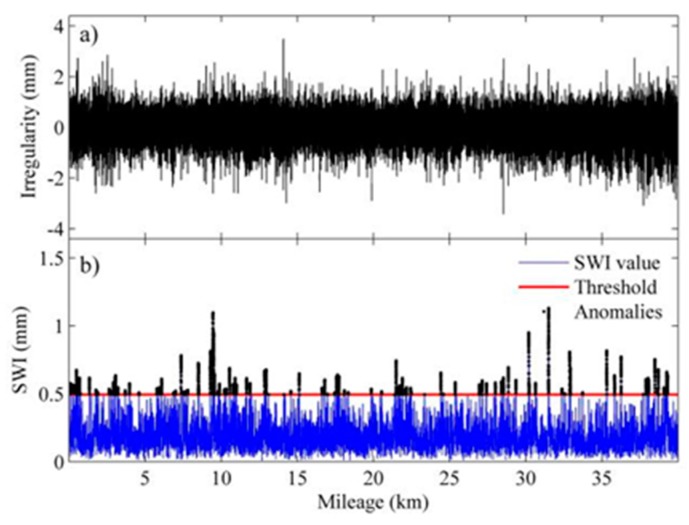
Track geometry and SWI of rail Section II: (**a**) waveform of track vertical irregularity; (**b**) SWI sequence and anomalies.

**Figure 15:**
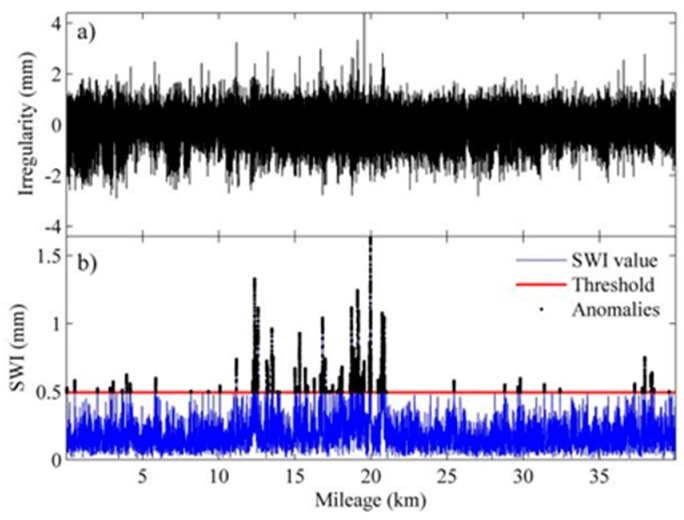
Track geometry and SWI of rail Section III: (**a**) waveform of track vertical irregularity; (**b**) SWI sequence and anomalies.

**Figure 16:**
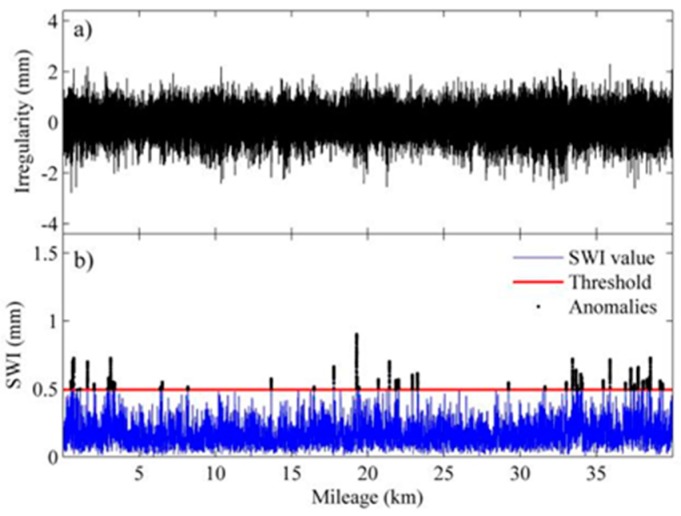
Track geometry and SWI of rail Section IV: (**a**) waveform of track vertical irregularity; (**b**) SWI sequence and anomalies.

To verify the proposed detection method, we chose four sections of an HSR line with warped slab(s) for the comparison of identification results by the proposed method and the visual inspection results. The length of each section is 40 km with 24,806 slabs in total. The upper panels of Figure 13, Figure 14, Figure 15 and Figure 16 show the waveforms of vertical irregularity data (after position synchronization) corresponding to the four sections collected by No. 0 CIT. The SWI sequences obtained with the proposed algorithm as well as those SWI values exceed the threshold are plotted in lower panels of Figure 13, Figure 14, Figure 15 and Figure 16. Segments with warped slabs are detected, and the severity of SWD is shown clearly by the SWI values. The detection results of the two sections are summarized in Table 3, which lists the number of slabs being detected as warped slabs. After comparison with the actual slab condition confirmed by visual inspection, the false alarms and the missed alarms are obtained and are listed in the last two columns of Table 3.

It can be concluded that: (1) more warped slabs in Sections II and III are detected than in Sections I and IV, which matches the defect database (record of regular visual inspection); (2) compared with SWI waveforms, the waveforms of geometry data of all these sections present similar patterns, which indicates that it is difficult to identify warped slab directly from geometry data, and the need for the proposed SWD identification method is self-evident; (3) altogether 160 slabs are confirmed as warped slabs by visual inspection, of which 151 (94.4%) are successfully detected by proposed method, while of the remaining 24,646 slabs, only 32 are falsely realized as warped slabs, so the overall recall of the proposed detection method is over 94%.

## 5. Conclusions

The temperature-induced SWD is a critical structural stability problem for HSR slab track systems in operation. It can cause excessive track irregularity and intensify the vibration of the vehicle–track system, leading to a reduction in ride comfort and operation safety. Compared with deterioration analysis and effect characterization of structural damages for slab track, less attention has been paid to the development of a sophisticated detection method of such deformation. Since the deterioration of rail infrastructure can be manifested in track geometry changes, it is reasonable to detect deformation by tracking the change of track geometry condition.

Based on this idea, this study intends to develop a reliable automated procedure for SWD detection in light of track geometry data. The main task of this study is to locate warped slabs as well as to translate the problem of SWD into a meaningful numerical rating. The ultimate aim is to provide certain technical support for a slab track maintenance and repair program. To this end, we first eliminate milepost error through position synchronization based on GRA to enhance the credibility of SWD detection and localization. The track geometry data of a typical track section with warped slabs are then chosen for SWD feature analysis. The samples are collected from different inspection runs from winter to summer of a year (one run per month) so that the change of track geometry condition with time can be observed, and the feature of temperature-induced SWD can be seen clearly on the waveform of track vertical irregularity. In frequency-domain analysis, the PSD of geometry data of track segment with warped slabs has a single peak at wavelength equal to slab length, which further proves the feasibility of using track geometry data for SWD detection. Considering that the features of warped slabs on track geometry data exhibit as localized anomalies which are non-stationary, the severity of SWD is quantified by establishing an index named SWI based on DWT. Subsequently, a threshold (limit) is placed using a pattern classifier which is formulated in terms of minimum error rate classification, thereby we finally can identify the warped slabs and assess the slab status based on the track geometry data only.

The present study allows for more accurate repair decision making for HSR slab tracks and further improvement of the durability and serviceability of the slab. However, to better facilitate the implementation of track geometry data-orientated slab maintenance works, the correlation between the amplitude of the SWD and SWI value needs further discussion. In the future, when more measured data of slab deformation are available, the SWI, as well as the threshold in judging the slab status, can be further optimized with the use of advanced pattern recognition techniques and machine learning methods, which would also enhance accuracy in detection and severity quantification of SWD. Apart from the improvement of signal processing method, future works should also focus on the research and development of novel NDT-based slab inspection devices, which can provide detailed inspection for every single slab, so that a multi-level slab detection scheme can be formed—the warped slabs are first located (out of tens of millions of slabs) with track geometry data, then a specific device or NDT technique is used to confirm the SWDs and draw the pattern of the specific defects behind them.

## Figures and Tables

**Table 1:** Procedure of SWI calculation.

**Input**: **s_r_**—track vertical irregularity data, **m_r_**—the milepost sequence of the irregularity data (having the same size as **s_r_**); *Th*—Threshold ^1^ of SWI, *n*—sample size of geometry data subjected to each step of SWI calculation, *fs*—spatial sampling frequency.	
Output*: **SWI**—SWI sequence, **Wa**—sequence recording SWIs that exceed threshold, and **ma**—the sequence of mileposts corresponding to **Wa**.	
**Step**	**Procedure**
1	Data preprocessing: Position synchronization based on grey relational analysis (GRA) to obtain corrected irregularity data **s** and corresponding milepost **m**.
2	Define *N* = length (**s**), *i* = 1; define SWI sequence: **SWI** = zeros (1, *N* − *n +* 1) and the corresponding milepost sequence **mw** = zeros (1, *N* − *n* + 1); define **Wa** = [] and **ma** = [].
3	Define the segment for SWI calculation: **s**^(*i*)^ = **s** (*i*:*i* + *n* − 1).
4	Conduct SWI calculation with Equation (6) to obtain *W_i_* as the SWI value corresponding to **s**^(*i*)^.
5	Conduct **SWI**(*i*) = *W_i_* and **mw**(*i*) = **m**(*i* + (*n* − 1)/2).
6	Check if *W_i_* > *Th*: if yes, go to **Step 3**; otherwise, go to **Step 4**.
7	Conduct **Wa** = [**Wa**, *W_i_*], **ma** = [**ma**, **mw**(*i*)].
8	Conduct *i* = *i* + 1.
9	Check if *i* > *N* − *n* + 1: if yes, **end procedure**; otherwise, repeat **Step 3**.

**Table 2:** Information of track slabs used as training data for setting threshold.

	In Healthy State	With Warping Deformation
Number of slabs with on-site inspection record	120,110	625
Number of inspection runs of CIT	104	104
Range of atmospheric temperatures *	−4 to 40 °C	21–40 °C

*: The atmospheric temperatures are recorded by the meteorological department.

**Table 3:** Summary of detection results for track slabs based on track geometry data.

**Sections**	**Total Slabs**	**Anomalies ^1^**	**False Alarms ^2^**	**Missed Alarms ^3^**
Section I	6202	33	6	2
Section II	6201	61	12	4
Section III	6202	60	10	3
Section IV	6201	29	4	0
Total	24,806	183	32	9

Note : ^1^ The number of slabs that are detected as warped slabs based on geometry data; ^2^ The number of healthy slabs that are detected as warped slabs based on geometry data; ^3^ The number of warped slabs that are realized as healthy slabs based on geometry data.

## Acronyms

The following acronyms are used in this manuscript:

CA mortar	Cement-emulsified asphalt mortar	CBM	Condition-based maintenance
CIT	Comprehensive inspection train	CRTS	China Railway Track System
DIC	Digital image correlation	DWT	Discrete wavelet transform
EMU	Electric multiple unit	GIA	Grey incidence analysis
GPR	Ground-penetrating radar	GRA	Grey relational analysis
HSR	High-speed rail	HSTGC	High-speed track geometry car
NDT	Non-destructive test	PDF	Probability density function
PSD	Power spectrum density	RMS	Root mean square
SWD	Slab-warping deformation	SWI	Slab-warping index
TGC	Track geometry car	TGC	Track geometry index
TQI	Track quality index	TRI	Track roughness index
